# Vaccines as Epidemic Insurance

**DOI:** 10.3390/ijerph14111304

**Published:** 2017-10-27

**Authors:** Mark V. Pauly

**Affiliations:** Department of Health Care Management, The Wharton School, University of Pennsylvania 208 Colonial Penn Center, 3641 Locust Walk, Philadelphia, PA 19104, USA; pauly@wharton.upenn.edu; Tel.: +1-215-898-2838

**Keywords:** insurance, vaccines, pandemics, risk, vaccine pricing, mutual insurance

## Abstract

This paper explores the relationship between the research for and development of vaccines against global pandemics and insurance. It shows that development in advance of pandemics of a portfolio of effective and government-approved vaccines does have some insurance properties: it requires incurring costs that are certain (the costs of discovering, developing, and testing vaccines) in return for protection against large losses (if a pandemic treatable with one of the vaccines occurs) but also with the possibility of no benefit (from a vaccine against a disease that never reaches the pandemic stage). It then argues that insurance against the latter event might usefully be offered to organizations developing vaccines, and explores the benefits of insurance payments to or on behalf of countries who suffer from unpredictable pandemics. These ideas are then related to recent government, industry, and philanthropic efforts to develop better policies to make vaccines against pandemics available on a timely basis.

## 1. Introduction

In the face of threats from communicable infectious diseases such as Ebola or the Zika virus, public health policymakers and philanthropists have turned their attention to ways to deal with such risks. One obvious thought is that, if a preventive vaccine could be developed and administered to populations at risk, the harm—both to health and wealth—from epidemics could be greatly reduced. A popular metaphor is that the development of such a vaccine, were it to be successful, would constitute “insurance” against the harms from any future outbreaks [1]. In this paper, I want to look at vaccine development and diffusion from an insurance perspective, and see what that perspective has to say about the efficiency and feasibility of vaccine development programs. My focus will be on the risk and insurance for firms, philanthropies, or governments concerned with having vaccines available if a pandemic strikes; I will not explore in detail insurance aspects of patient access and financing, nor will I deal with other dimensions of responses to pandemics such as the isolation or stockpiling of vaccines. I will also not deal with efforts to make “surge funding” available at the outbreak of an epidemic, the target of the World Bank’s “Pandemic Emergency Facility” (PEF), a joint effort with Germany and Japan and the world’s largest reinsurance funds [2]. Instead, my focus is on making sure that an effective vaccine is available and ready to go when the emergency funding is triggered.

Insurance is an arrangement in which a population at risk of some future loss makes a fixed certain payment—the premium—in return for financial protection against losses should they occur. If no loss occurs, the risk-averse person who “lost” the premium is still better off than experiencing the loss while paying no premium. That fixed premium payment can be used in two ways: (1) to compensate losers financially after a loss, holding the loss prospect constant and/or (2) funding ways to prevent or mitigate the loss, so that large financial losses are less common. I assume that the insurance metaphor for vaccines represents the second type (sometimes called “self-protection”). Yet, does the metaphor work—and are there other insurance alternatives to deal with this problem? In this paper, I will argue that not only does this metaphor limp much more than usual, but there are insurance alternatives that can replace or supplement investment in developing vaccines for uncertain future epidemics that should be considered.

## 2. A Little Insurance Theory

Insurance can protect agents (including firm owners) who are risk averse against financial losses in certain circumstances. For a harmful or loss-producing event to be ideally insurable, it should have the following characteristics: it should be generated by risky but independent events, the administrative cost of arranging insurance should be modest, and the provision of insurance should not change the insureds’ behavior. From this viewpoint, a program to pay real resources to develop vaccines for uncertain future diseases has some issues.

First, virtually by definition, an epidemic of contagious disease is the antithesis of independent harmful events. Instead, when an epidemic breaks out, harm to one person is surely correlated with harm to many other people. This means that “epidemic insurance” for a single type of viral threat cannot take advantage of the law of large numbers which allows a fixed payment to come as close as we like to offsetting the harm from some adverse event.

The risk in this case is that payment will be made to develop a vaccine against a possible disease which is never needed to protect against a large scale epidemic of that disease. This phenomenon (possible after-the-fact loss) alone is common to all insurances; many people will pay premiums and collect nothing—but in every time period some people will collect much more than they paid in, and that is the benefit from the insurance. In the case of developing an expensive vaccine that is never used, there is no such offsetting benefit.

There could be an insurance-like gain if the program is to develop a portfolio of vaccines against many different types of disease, so that the probability that some of those vaccines are going to be of benefit is high. The important point in this case is that the value of the program is not based on the worst case scenario in which every vaccine is needed, but instead is based on the average or expected prospective usage of each vaccine, potentially adjusted upwards to reflect people’s willingness to pay to avoid risk.

Depending on difference in timing between when the vaccine is administered and when an epidemic breaks out, it may or may not be possible to know if the vaccine provided any benefit. If the vaccine is administered well in advance of identifying an epidemic and immunity then prevents any epidemic from occurring, we will never know if the vaccine provided protection or not. If it is kept in reserve and only administered once some cases suggesting the potential of an epidemic have occurred, at least we will know if it mattered.

## 3. Funding and Motivating the Development of Vaccines against Future Epidemics

Interest by foundations and philanthropists in advanced development of vaccines against future epidemics has been stimulated by the challenges posed both by the Ebola and Zika viruses. Obviously, commercial interest in investing in such development is limited by the poverty of citizens in the countries where they first strike. There is also a problem in generating a perception of need for a drug to treat a threat which, in the words of Dr. Anthony Fauci, director of the National Institute of Allergy and Infectious Disease, “hasn’t happened yet” [3].

Some nonprofit entrepreneurs have taken steps to develop organizations to perform the research and testing functions. For example, a former GlaxoSmithKline (GSK) scientist, who worked on developing an Ebola vaccine that eventually was not needed, has made efforts to create an entity to work on vaccines in a former GSK facility but has yet to secure adequate funding (beyond an apparently favorable rental arrangement with GSK). The plan is to invent vaccines and carry their development through Phase I and Phase II trials, with the expectation that the outbreak of an epidemic will provide the opportunity, the motivation, and influence at the U.S. Food and Drug Administration (FDA) to move the relevant vaccine through Phase III trials.

Such an effort, as well as others to be discussed below, provide the context for asking and discussing several key questions about vaccine development for prospective epidemics:

1. Does the firm doing the development need to be non-profit or can there be a useful opportunity for a commercial firm?
2. What revenues should vaccines actually launched in the case of an epidemic generate—whether those revenues are covered by foundation grants, public or private insurance, or sales in the pharmaceutical market?
3. Can conventional insurance assist in this development, given the interest of some commercial reinsurance firms in offering products of value when epidemics strike? These three key considerations will be discussed in what follows.

## 4. For Profit or Nonprofit?

If a vaccine was developed by a profit maximizing firm that was able to secure both patent protection and FDA-granted exclusivity, the concern is that the price or revenue the firm would seek would make the vaccine unaffordable for those who need it if an epidemic strikes. Indeed, the possibility of “price gouging” in the face of a sudden surge in demand motivates much of the consideration of alternative development arrangements. The fear is that, if left to the private market, either no vaccine will be developed when an epidemic strikes because firms correctly calculate that patients do not have enough income to allow them to pay prices that will cover development costs or, if a vaccine is developed, the surge in demand will lead to prices and revenues that overcompensate investors by overcharging buyers.

The idea that prices would be set so high that no one would be able to afford a vaccine that is being developed is nonsensical, since the firm can only make profits by setting the price low enough that some buyers are willing to pay it. However, it is true that, with government guarantee of monopoly, the price will be set higher than the cost of manufacturing the vaccine, and potentially higher than the R&D costs. A nonprofit firm would not pose this threat, but neither would a for profit firm if a tight contract to develop an effective vaccine and make it available at a prespecified price could be written. Ultimately, the form of ownership depends on which type of firm can be more closely monitored. The provision of vaccines that could not be sold at a profit depends on donors’ willingness to make up any shortfall between revenues or prices and costs.

The more important point is that, whether a firm is for profit or nonprofit, it cannot be sustained if it makes losses. Regardless of ownership type, expected revenues must be high enough to cover expected costs. The main dividend from nonprofit status is greater assurance that revenues will not exceed costs by a large amount—but neither ownership form can survive if revenue falls short of cost (of R&D and production).

## 5. How Much Is Enough Revenue?

As already noted, a firm that needs (at minimum) to cover its costs from outside revenues will be challenged if it is possible that a vaccine that is expensive to develop and test is not needed. Either the cost of developing vaccines that turn out to be unneeded needs to be funded, or the revenues from those vaccines for which there is need because an epidemic breaks out will have to be high enough to cover past investment in vaccines for other diseases that did not occur and so failed to generate enough revenue to cover their R&D costs. There are two reasons why the development of a vaccine might not cover its cost. Most obviously, the disease for which it is to be used may fail to appear in a reasonable period of time. Less obviously, the disease may appear but if there is timely and well planned use of the vaccine in the initially small population affected, the epidemic may be stopped in its tracks—which is good news except that it means that not much vaccine will be sold. To cover these losses, “profiteering” in terms of high pricing for a vaccine that is used after a pandemic is in progress is to be expected and accepted as a way of providing sufficient funding for R&D investment of uncertain payoff and incentives to undertake that R&D.

This point—coping with potential losses from developing vaccines that turn out to be unneeded in large quantities—is the missing link in the vaccines-as-insurance metaphor. It is not enough to have vaccines when they are needed; instead, pricing or revenues for the global process of developing an inventory of approved vaccine concepts and materials must also be supported in some fashion. Accomplishing this task is something that actual insurance can help with. An organization that is large enough to invest in a portfolio of vaccines may itself be able to cover the wasted costs of vaccines that are not needed by making profits on those that are—but it would have to be permitted to do so.

## 6. Insurance for Product Development Costs

One solution to this problem is to imagine that a large insurer (e.g., Lloyds or Munich Re) can estimate the risk that a vaccine under development will not be needed, and then sell insurance which pays off when that (non)event occurs. (After all, Lloyds insured the prize offered by a company’s ad campaign against discovering the Loch Ness Monster) [4,5]. The cost of insurance could and would be built into the firm’s total cost, so it would charge higher prices to cover it—but then those higher prices would go not toward excessive profits but just to cover insurance costs.

Using insurance to hedge the risk that a product under development will have zero demand because its target never causes an epidemic may be an efficient and acceptable way to motivate upfront investment by non-philanthropic or philanthropic capital, since it avoids the possibility of wasting investment on a product that is never needed. A guarantee that risky investments will not lead to losses may attenuate incentives for the investing entity to be cautious in taking risk—so called “moral hazard”—so either partial risk sharing or serious supervision of investment choices may need to be built into the contract.

There is an alternative device that may help as well—mutual insurance. Suppose there are many firms developing different vaccines, whose funders have different views on the likelihood of an outbreak of any epidemic. If they nevertheless agree on a set of epidemics that are regarded as equally likely, they can set up a mutual insurance arrangement, with an initial pledge of funding close to the highest estimate of epidemic likelihood. Then, if fewer epidemics materialize so less insurance benefits need to be paid out of the pool than the worst-case expectation, the pool can pay out any surplus as a dividend.

This arrangement should be equally attractive to optimists and pessimists, those who think an epidemic unlikely and those who are sure many will occur, since the former will expect to get money back (with interest) and the latter will be grateful for insurance coverage.

## 7. Spot Insurance and Vaccines

Current policy developments have not, to our knowledge, directly involved vaccine makers. Instead, they have taken the more direct route of developing mechanisms to make cash available to countries hit by an epidemic. Furthest along is a project in Africa, under the heading of “African Risk Capacity (ARC)” [6]. ARC is part of a larger and longer running effort to make insurance for protection against climate disasters (drought, windstorms) available to farmers and other businesses in Africa. The intent is to develop a vehicle, funded by contributions from African governments and philanthropic organizations, that would make available, as a sovereign insurance product, the payment of timely early funds, so governments hit by an epidemic can fund an immediate response (rather than wait for the slower process of the application for assistance and mobilization of resources). This product is planned to be rolled out sometime in 2018. The development of methods to detect early signs of disease and specify trigger indicators that can start the flow of funds, which in turn can fund pre-planned response activities, is daunting but underway nonetheless.

Timely availability of effective and approved vaccines to vaccinate at risk populations is clearly one of the responses that should be sought if possible. Here we have insurance that pays for vaccines that do turn out to be needed (as opposed to paying for ones that do not). How would the availability of this type of insurance fit into the vaccine development process? The key issue is the price to be paid. How large does it have to be to motivate supplying organizations to develop the vaccines that can be used in pre-planned responses? The answer to this puts us back on familiar ground—the payment must be enough to cover not only the R&D costs of the vaccine that will be used, but also to cover as well the cost of providing the “option” of having a vaccine available that is never used. Determining that price will be a challenge, for some of the reasons already noted.

## 8. Some Serious and Practical Challenges

So far we have the optimistic outlook that there are two kinds of insurances that may make the development and timely use of vaccines against potential pandemics feasible—either pay in the event a vaccine is not needed, or pay in the event that it is. However, there are (at least) three problems with the practical operation of such schemes that need to be solved.

What is the trigger? Any insurance needs to specify the event or circumstances that trigger the payment of benefits, and ideally that event should be outside the control of those insured. Drought insurance, for example, uses data on rainfall (or the absence thereof) to trigger payments. What are the analogues for insurance against unneeded vaccines or insurance against actual pandemics? In both cases, there is objective information—the length of time a developed vaccine has remained “on the shelf”, or the frequency of cases of the disease. Yet, in both cases the insured entity might be able to manipulate the trigger—by choosing to develop vaccines against diseases that are unlikely to occur, or by allowing some cases of a contagious disease to go without treatment until the trigger number is reached. This distortion of incentives by insurance is a kind of moral hazard, and needs to be detected and prevented to the extent that is possible.

Where is the data for epidemic insurance? There is extensive data on the frequency of deaths at various ages stretching back decades. If insurers are willing to assume that the future will be similar to the past, such data can make it easy to estimate the premium or contribution that will cover the expected payoff. However, the availability of data to predict the frequency and severity of epidemics is both more limited and more specific (to the kinds of epidemics that occurred) than rainfall data. Insurers therefore will be somewhat apprehensive in offering coverage since they do not have the data that allows them to price it in a way that can be justified as (at least) likely to cover claims costs. This is not necessarily a fatal flaw—some insurers, such as Lloyds, are willing to offer cover for unique events, and mutual insurance is a way of dealing with uncertainty about loss probabilities if it is accompanied by certainty about which risks are the same.

Note, however, that there are some differences between the two insurances in the kinds of information they need. After-the-fact insurance like that from the ARC needs to estimate both the existence of a contagious disease and the amount of harm it will do. However, the premium for an insurance against a vaccine’s chances of not being needed can be based solely on whether the disease it protects against breaks out and the (average) cost of R&D for a vaccine; it does not need precise data on the number of patients affected or at risk beyond establishing that there are “many.”

What if an epidemic turns into a pandemic? This question concerns the availability of capital to an insurer covering risks that are correlated. It is most severe for insurance against the cost of an epidemic that emerges, and occurs if the volume of losses to be covered is so great that the insurer does not have enough in reserves to pay for them. First line insurers like ARC will buy reinsurance to protect against this event, usually by specifying a maximum total amount they will pay out of their own resources before a reinsurer becomes liable. If epidemics, such as drought, only affect a limited geographic area at the time, there can be risk pooling across countries or areas within a continent or around the world. It will make sense for a reinsurer to write limited business in many different geographic locations to try to cope. Also, the worldwide pool of total capital is large enough that by pooling with other risks reinsurers it may be able to obtain substantial diversification. Fatally catastrophic bonds, where interest and principal are foregone if a pandemic strikes, is another way to tap the worldwide capital pool. Very high losses for product insurance are unlikely to occur unless contagious disease totally disappears. That is, it is unlikely that no vaccines will be needed for a substantial period of time.

How closely should insurers monitor the insureds’ loss mitigation efforts? The ARC imagines that potential insured countries will work with it to establish efficient quick response procedures and other ways to reduce expected losses, by steps that either lower the probability that a disease strikes or the damage that it does. An insurer can give conditions that the offering of insurance will depend on the extent of pre-planned responsiveness. Alternatively, insurance may be offered but its premium surcharged if prevention efforts are judged to be below par.

## 9. What Is Needed to Move Forward

The most obvious necessity for making progress in reducing the risk of harm from epidemics is a recognition that something needs to be done, even when the world is not at present in the throes of some serious challenge. The cycle of a “fire drill” when a new infectious agent is identified—whether it ultimately leads to a harmful epidemic like Ebola or turns out to be false alarm like bird flu—followed by no activity as the threat recedes in time, until the next threat develops, may be human nature, but it should be avoided by planning ahead. That planning should move on two fronts: (1) the development of materials and methods to reduce the chances of harm or mitigate actual losses and (2) the simultaneous development of and coordination with financial insurance to create tools for mitigation and offset the losses of harms that can never be completely prevented.

In both cases, enthusiasm should be tempered by cost-benefit analysis: can the proposed vaccine be developed and deployed at a reasonable cost, and how much does an insurance pool cost to assemble and administer? Here again, the daunting task of estimating the probability of future harms will be important and difficult.

A challenge potentially easier to deal with is that of finding capacity to manufacture vaccines against an epidemic. If an entity is willing to bear the costs of regulation-required clinical trials and storage, there could be a stockpile of vaccines in case of emergency. Rules might be modified to permit the use of vaccine that has passed Phase II trials and to monitor for the less frequent harms that might occur when it is used on a population basis. Or, there could be mothballed capacity set aside for this purpose. Another possibility is to stockpile other medicines whose production facilities can be diverted to vaccine production in the event that a disease reaches epidemic level. In each case, the relevant variables are the cost of each strategy and their ability to deliver protection on a timely basis.

Dr. Rino Rappuoli of GSK has proposed a program in which vaccine development against 30 diseases would be funded by all governments. This is a large enough portfolio that the risk of high losses from a vaccine that turns out to be unneeded can be mitigated. The remaining challenge here is likely to be willingness to pay, which in turn will depend on presenting evidence of net benefit from a program at this scale. There is also the issue of centralized planning by governments (which ones?) versus use of a market-based system where government fosters the development of insurances for firms and affected populations by setting or permitting adequate rewards to risk taking as opposed to government estimating and taking the risk itself.

## 10. Current Efforts to Reduce Risk and Increase Preparedness

The challenge of having vaccines available on a timely basis received a major boost earlier this year when Bill Gates announced at the Davos meeting that his foundation, along with the Wellcome Trust and the governments of Norway and Germany, were will willing to make several hundred million dollars of resources available to develop a mechanism to meet it. The effort is labeled CEPI (Coalition for Epidemic Preparedness Innovations) [7].

The immediate stimulus to CEPI was a series of reports from an expert panel (the expert panels were the WHO Ebola Interim Assessment Panel, Harvard-LSHTM Independent Panel, US National Academy of Medicine and the UN Secretary General’s High Level Panel) on Ebola that lamented the absence of a vaccine when the epidemic began and then the shelving of two vaccines, one from Merck and one from GSK, as the epidemic ended just as those products were emerging and receiving regulatory approval [8,9,10].

What exactly an entity like CEPI should do in terms of financing and insurance or risk reduction is purposely vague in the expert reports and statements. Technologically, the goal seems to be to develop a platform adaptable to the DNA and RNA of whatever specific virus emerges so that there can be a quick response, along with stockpiling hundreds of thousands of doses by Gavi, the international organization for vaccine policy. The intent seems to favor nonprofit organizations, but without recognition that uncertainty makes it very hard to hit the goal of breakeven precisely, and with almost no consideration of the prices or revenues that the entity doing the development might receive. There is some discussion of the partial insurance approach of gainsharing, but little else—hopes for ideal technology development and better planning of responses from a public health perspective put financing, incentives, and financial sustainability issues to the side.

The opportunity for insurance of several sorts to contribute to the solution should not, however, be overlooked. Several large reinsurers have indicated an interest in helping; in addition, the drug firms that were disappointed to arrive too late and the scientists who lamented the cancelation just as they were on the verge of fulfilling their task all are eager to contribute. Examining how real insurance can contribute to the metaphorical insurance represented by vaccines and to the greater goal of protecting populations, both in developing and developed countries, from avoidable risk seems promising.

## 11. Conclusions

Market insurance has a potential role to play in helping to assure that effective vaccines are available when a pandemic strikes.
